# Simultaneous RNA and DNA Adductomics Using Single Data-Independent Acquisition Mass Spectrometry Analysis

**DOI:** 10.1021/acs.chemrestox.3c00041

**Published:** 2023-08-11

**Authors:** Giulia Martella, Nisha H. Motwani, Zareen Khan, Pedro F. M. Sousa, Elena Gorokhova, Hitesh V. Motwani

**Affiliations:** † Department of Environmental Science, Stockholm University, Stockholm SE-106 91, Sweden; ‡ School of Natural Sciences, Technology and Environmental Studies, Södertörn University, Huddinge SE-14189, Sweden; § Department of Materials and Environmental Chemistry, Stockholm University, Stockholm SE-106 91, Sweden

## Abstract

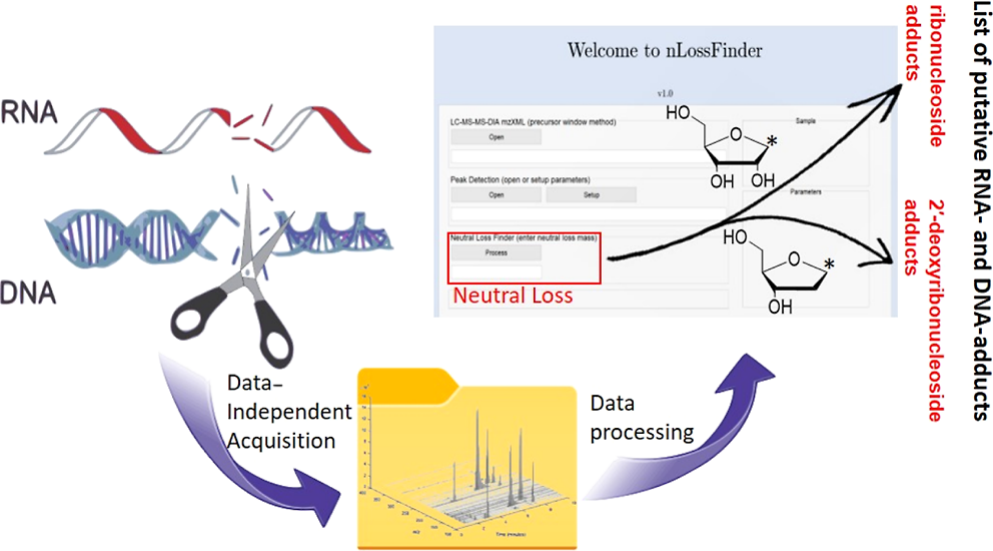

Adductomics studies are used for the detection and characterization of various chemical modifications (adducts) of nucleic acids and proteins. The advancements in liquid chromatography coupled with high-resolution tandem mass spectrometry (HRMS/MS) have resulted in efficient methods for qualitative and quantitative adductomics. We developed an HRMS-based method for the simultaneous analysis of RNA and DNA adducts in a single run and demonstrated its application using Baltic amphipods, useful sentinels of environmental disturbances, as test organisms. The novelty of this method is screening for RNA and DNA adducts by a single injection on an Orbitrap HRMS instrument using full scan and data-independent acquisition. The MS raw files were processed with an open-source program, *nLossFinder*, to identify and distinguish RNA and DNA adducts based on the characteristic neutral loss of ribonucleosides and 2′-deoxyribonucleosides, respectively. In the amphipods, in addition to the nearly 150 putative DNA adducts characterized earlier, we detected 60 putative RNA adducts. For the structural identification of the detected RNA adducts, the MODOMICS database was used. The identified RNA adducts included simple mono- and dimethylation and other larger functional groups on different ribonucleosides and deaminated product inosine. However, 54 of these RNA adducts are not yet structurally identified, and further work on their characterization may uncover new layers of information related to the transcriptome and help understand their biological significance. Considering the susceptibility of nucleic acids to environmental factors, including pollutants, the developed multi-adductomics methodology with further advancement has the potential to provide biomarkers for diagnostics of pollution effects in biota.

## 1 Introduction

Ribonucleic acid (RNA) and deoxyribonucleic acid (DNA) are nucleotide polymers carrying genetic information essential for life. These nucleic acids are covalently adducted with various chemical groups, collectively called the adductome. As RNA and DNA have different roles in storing genetic information and transcript processing, it is essential to understand adduct formation and the mechanisms involved in both nucleic acids. Moreover, adduct formation and dysregulation on the single-stranded RNA may be more responsive to environmental stimuli than the rigid double-stranded DNA molecule and, thus, provide more information on the acute stressors and biological adaptions to handle them.

**Figure 1:**
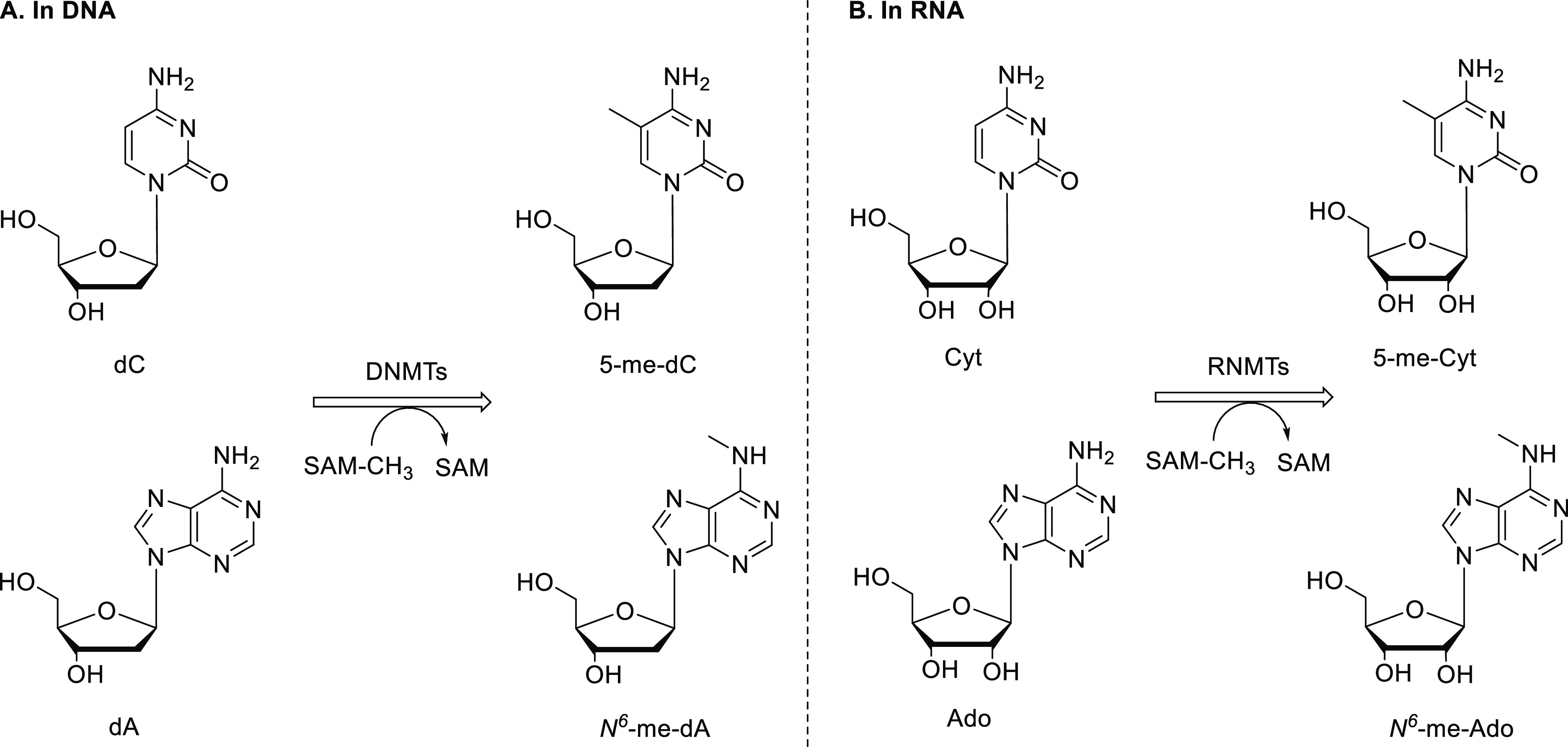
DNA- and RNA-methylation catalyzed by DNA methyltransferases and RNA methyltransferases, respectively, using *S*-adenosylmethionine as the methyl donor.

Understanding the formation and functions of all nucleic acid modifications and their measurement is essential for deciphering the full adductome. Methylation (Figure 1) is one of the most abundant modifications of nucleic acids, whereby the methyl group is adducted on the nucleobase (e.g., cytosine or adenine), resulting in methylated adducts of RNA or DNA.^1−5^ While only a few methylated DNA adducts are identified, nearly 70 types of RNA methylation are known for all RNA species, including transfer RNA, ribosomal RNA, and messenger RNA.^3,6^ The methylated adducts can be further oxidized, including ten-eleven translocation (TET) mediated oxidation of 5-methyl-2′-deoxycytidine to 5-hydroxymethyl-2′-deoxycytidine.^7,8^

When considering the global adductome, in addition to the epigenetically modified DNA or post-transcriptionally modified RNA, chemical modifications on the nucleophilic sites of the nucleobases [i.e., *N*- and *O*-atoms; see Motwani et al. 2018^9^ for the nucleophilic sites] can also result from binding to genotoxic (electrophilic) reactive compounds or metabolites by a nucleophilic substitution mechanism. These types of adducts are primarily studied in DNA [e.g., Balbo et al. 2014; Hemeryck et al. 2015; Motwani 2020; Motwani et al. 2020^10−13^], but a few studies^14−16^ have shown such nucleophilic substitution binding of electrophilic compounds to RNA as well.

Adductomics is an emerging-*omics* technique that includes screening and identifying chemical modifications on biomolecules, e.g., RNA or DNA. Liquid chromatography (LC) coupled to tandem mass spectrometry (MS/MS) is well-recognized for the structural characterization and measurement of selective modifications on RNA and DNA that other methods, such as bisulfite sequencing (to determine the pattern of RNA or DNA methylation) and ^32^P-post labeling (to identify exposure to bulky genotoxic compounds), cannot obtain. High-resolution mass spectrometry (HRMS) using Orbitrap instrumentation has advanced the field of adductomics because the accurate masses obtained from the adduct analytes improve the resolving power to detect adducts in complex mixtures. In addition, the high-resolution accurate mass (HRAM) data enable a more precise determination of the elemental composition of the adducts, which can improve the identification of possible structural modifications on the nucleobases.

HRMS-based DNA adductomics has been employed to detect adducts in various cell types, such as liver tissue,^12^ lung and esophagus,^17^ exfoliated urinary cells,^18,19^ and crustaceans.^20,21^ HRMS acquisition methods applied in metabolomics or proteomics studies are being adapted for adductomics approaches, e.g., data-independent acquisition (DIA) and data-dependent acquisition. Concurrently, software-assisted tools are being developed for DNA adductomics to process large and complex MS data files generated by these acquisitions.^22−24^ We recently developed a graphical user interface (GUI; MATLAB) program, *nLossFinder*,^22^ to detect 2′-deoxyribonucleoside adducts originating from DNA digests. This program was developed for untargeted screening of DNA adducts analyzed by LC-HRMS/MS on an Orbitrap employing full scan (FS) merged with DIA sequential precursor windows method. The detection of 2′-deoxyribonucleoside adduct ions by *nLossFinder* is primarily based on the characteristic neutral loss of the deoxyribose moiety (dR, 116.0474 Da) from the adduct with high mass accuracy. However, the adductomics approach based on capturing the neutral loss invariably does not detect certain classes of adducts, such as cross-linked adducts (e.g., DNA–DNA, DNA–RNA, or DNA–protein),^25^ depurinated adducts,^26^ and adducts on the phosphate backbone of the DNA.^27^

Compared to DNA adductomics, RNA adductomics is a relatively less explored field, even though it has been suggested that RNA modifications drive many biological processes.^15,25,28−30^ We advocate that nucleic acid modifications can be used as biomarkers to identify exposure effects at the genome and transcriptome level in biota, thus providing a tool in biological effect monitoring.^20,21^ When characterizing the DNA nucleosides in FS-DIA runs on the Baltic amphipods, *Monoporiea affinis*, small benthic crustaceans, we detected two relatively intense peaks in the extracted ion chromatogram (EIC) for the protonated 2′-deoxyguanosine (dG) parent ion using a narrow mass range (±5 ppm).^22^ The additional peak, not belonging to dG, originated from RNA and corresponded to the ribonucleoside adenosine (Ado); the protonated molecular ions of dG and Ado have the same mass, *m*/*z* 268.1040. This finding led us to investigate the possibility of the simultaneous characterization of the nucleic acids’ adductome by utilizing the archival nature of the FS-DIA approach. The objectives of this study were to characterize the nucleic acids’ adductome in the test organism and present a workflow for simultaneous RNA and DNA adduct identification and characterization. A proof of principle for the simultaneous multi-adductomics workflow was demonstrated by analyzing RNA and DNA adducts in the amphipods, *M. affinis*. This is the first multi-adductomics study employing a single MS run on the global RNA and DNA level to the best of our knowledge.

## 2 Materials and Methods

### 2.1 Chemicals and Other Materials

Guanosine (Guo), cytidine (Cyt), adenosine (Ado), uridine (Urd), 5-methylcytidine (5-me-Cyt), 5-methyluridine (5-me-Urd), *N*^*6*^-methyladenosine (*N*^*6*^-me-Ado), 2′-deoxyguanosine (dG), 2′-deoxycytidine (dC), 2′-deoxyadenosine (dA), thymidine (dT), 5-methyl-2′-deoxycytidine (5-me-dC), *N*^*6*^-methyl-2′-deoxyadenosine (*N*^*6*^-me-dA), 8-oxo-7,8-dihydro-2′-deoxyguanosine (8-oxo-dG), nucleosides test mix [containing cytidine (Cyt), guanosine (Guo), adenosine (Ado), uridine (Urd), inosine (Ino), 5-methylcytidine (5-me-Cyt), 2′-*O*-methylcytidine (2-*O*-me-Cyt), 3-methylcytidine methosulfate (3-me-Cyt), 7-methylguanosine (7-me-Guo), 1-methyladenosine (1-me-Ado), 5-methyluridine (5-me-Urd), β-pseudouridine (β-Urd), 2-thiocytidine dihhydrate (ThioC)], DNA from calf thymus (ctDNA) sodium salt, nuclease P_1_ from *Penicillium citrinum* (NP1), phosphodiesterase I from *Crotalus adamanteus* (snake) venom (SVPDE), alkaline phosphatase from *Escherichia coli* (AKP), ammonium acetate, ammonium bicarbonate, tris(hydroxymethyl)aminomethane (Tris-buffer, pH 7.4), zinc chloride, and formic acid were obtained from Sigma-Aldrich (St. Louis, MO). Chelex-100 resin was purchased from Bio-Rad (Solna, Sweden). All the solvents used were of HPLC grade. Experiments containing nucleic acids were carried out in DNA LoBind tubes, 1.5 mL (Eppendorf).

### 2.2 Sample Collection and Preparation

Amphipods, *M. affinis* (adult females, 4–6 mg individual wet mass), were collected from the Northern Baltic Proper within the Swedish National Marine Monitoring Program; the sampling details and contamination status are described elsewhere.^20,21^ Nucleic acids were extracted from individual animals by heating with Chelex-100 ion-exchange resin and enzymatically digested as described in Gorokhova et al. 2020.^20^ Further, aliquots from the digested 12 amphipods^20^ were pooled in a septum-sealed vial to a final volume of 300 μL and stored at −20 °C till analysis by LC-HRMS as described in Sousa et al. 2021.^22^ Blank samples were obtained with the procedure being repeated in triplicates but without any DNA/RNA.

### 2.3 Liquid Chromatography–High-Resolution Tandem Mass Spectrometry

A Dionex UltiMate 3000 LC device coupled to an Orbitrap Q Exactive HF mass spectrometer (Thermo Fisher Scientific, MA) with a heated electrospray ionization source in positive mode was used. The MS was run in FS/DIA mode, with a DIA window wideness of *m*/*z* 10 and ranging from *m*/*z* 200 to 350. The full MS scan was conducted at a resolution of 120 000, an automatic gain control (AGC) target of 3e^6^, a maximum ion injection time (IT) of 200 ms, and a scan range from 110 to 650 *m*/*z*. The DIA sequential precursor window method was set to a mass resolution of 60 000, an AGC target of 5e^5^, a maximum ion IT of 120 ms, a loop count of 16, and a scan range from 195 to 355 *m*/*z*, which was divided into 16 discrete *m*/*z* ranges with an isolation window of 10 *m*/*z* (200 ± 5, 210 ± 5, and up to 350 ± 5 *m*/*z*). All conditions, including mobile phase, column, gradient, and MS parameters for MS1 acquisition and MS2 DIA, are described in our previous work.^22^

### 2.4 Data Processing

If the *m*/*z* of a nucleoside adduct’s parent ion is known, e.g., calculated or observed for a known adduct, the accurate mass of the corresponding specific fragment ion (or nucleobase adduct ion) can be calculated using the following equations
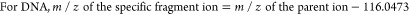

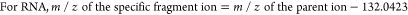


Data processing for detecting DNA adducts using the program *nLossFinder* is described in our earlier work.^22^ For data processing of RNA adducts, a list of 163 RNA modifications with accurate *m*/*z* of each molecular ion, [M + H]^+^, was exported from MODOMICS (http://genesilico.pl/modomics/modifications) to a .csv file. When obtaining the RNA modification list in MODOMICS, originating base was selected as “all bases”, and the selected chemical type of modification was *All*. The exported adduct list consisted of *Name*, *Short Name*, *Formula*, *Monoisotopic mass,* and *Protonated mass*; the list is shown in Supporting Information Table S1. The .csv file was then exported to TraceFinder software (V4.1, from Thermo Fisher Scientific Inc.), which was used to screen for the ribonucleoside adducts. The nucleoside adducts originating from RNA were considered as detected if the precursor ions were found in TraceFinder with a mass accuracy within 5 ppm of the theoretical mass and the presence of the ribose fragment ion (*m*/*z* 133.04954 ± 5 ppm) in the corresponding MS2 window.

Further, the HRMS data were processed by *nLossFinder* in MATLAB (MathWorks Inc., Natick, MA) for a nontargeted screening of RNA adducts with a mass range *m*/*z* of 200–350 of the nucleosides. This software was programmed^22^ to be coupled with an HRMS detection method that collects MS1 with an FS and MS2 with a DIA scan mode. For screening of DNA adducts, the deoxyribose neutral loss (dR, 116.0473 Da) was used,^22^ and in the present work for the screening of RNA adducts, the ribose (R) neutral loss corresponding to 132.0423 Da was employed. *nLossFinder* generated a list with putative nucleoside adducts from RNA based on peak matching between the MS1 ion and the MS2 ion generated from a neutral loss of 132.0423 Da. The mass tolerance was set to 5 ppm, and the minimum track points and the track missing points were 5 and 3, respectively. All other program settings were the same as in the software description.^22^ The adduct list from *nLossFinder* was converted to a .csv file and exported to TraceFinder. The adducts were again screened with Tracefinder for a second level of confirmation and considered as detected if the precursor ions were found in TraceFinder with a mass accuracy within 5 ppm of the mass imported from *nLossFinder* and the presence of the ribose fragment ion (*m*/*z* 133.04954 ± 5 ppm) in the corresponding MS2 window. The lists from the targeted approach (MODOMICS) and the nontargeted approach (*nLossFinder*) were combined to obtain each molecular ion’s peak area from EIC for all the detected nucleoside adducts, which were normalized to the peak area of Guo (peak area-adduct *100/peak area-Guo).

### 2.5 Identification

Preliminary identification of each RNA modification was based on comparison of the *m*/*z* [M + H]^+^ ion of the nucleoside adduct between that obtained from the high mass accuracy data of MS1 and the corresponding theoretical values calculated from the chemical formula obtained from MODOMICS using a 5 ppm accuracy window. The presence of MS2 *m*/*z* [(M – R)+H]^+^, nucleobase adduct fragment ion (±5 ppm), in the corresponding DIA window within the same chromatographic window of the parent nucleoside adduct ion in MS1, was used for confirmation. Further, the chemical structures of 5-me-Cyt, 5-me-Urd, *N*^*6*^-me-Ado, Ino, and 7-me-Guo were confirmed using authentic reference standards. Standard solutions of 5-me-Cyt, 5-me-Urd, and *N*^*6*^-me-Ado (each 100 fmol/μL) were prepared in deionized water, and Ino and 7-me-Guo (which were part of a mixture containing Cyt, Guo, Ado, Urd, Ino, 5-me-Cyt, 2-*O*-me-Cyt, 3-me-Cyt, 7-me-Guo, 1-me-Ado, 5-me-Urd, β-Urd, and ThioC in a range from 5 to 50 pmol/ μL) were diluted in deionized water in a range from 5 to 50 fmol/μL. The solutions were analyzed by LC-Orbitrap MS employing the same settings described above. The resulting HRMS data and retention time on the chromatogram were compared with the corresponding data obtained from the amphipod samples. Spiking of the amphipod samples with the respective standard solutions and comparison of LC-HRMS data with the non-spiked amphipod samples were performed to further confirm the identification of Cyt, Guo, Ado, Urd, Ino, 5-me-Cyt, 7-me-Guo, *N*^6^-me-Ado, and 5-me-Urd.

## 3 Results

### 3.1 Simultaneous Characterization of RNA- and DNA-Specific Nucleosides

The pooled, digested, amphipod RNA and DNA were analyzed by LC-HRMS/MS to characterize the 2′-deoxyribonucleosides dG, dA, dC, and dT (DNA) and ribonucleosides Guo, Ado, Cyt, and Urd (RNA). The detection was based on respective molecular and fragment ions by high mass accuracy and retention time (Table 1) and by the comparison with the respective standards (Figure S1). The specific fragment ions from each precursor nucleoside were detected in the respective MS2 DIA window (Table 1).

**Table 1:**
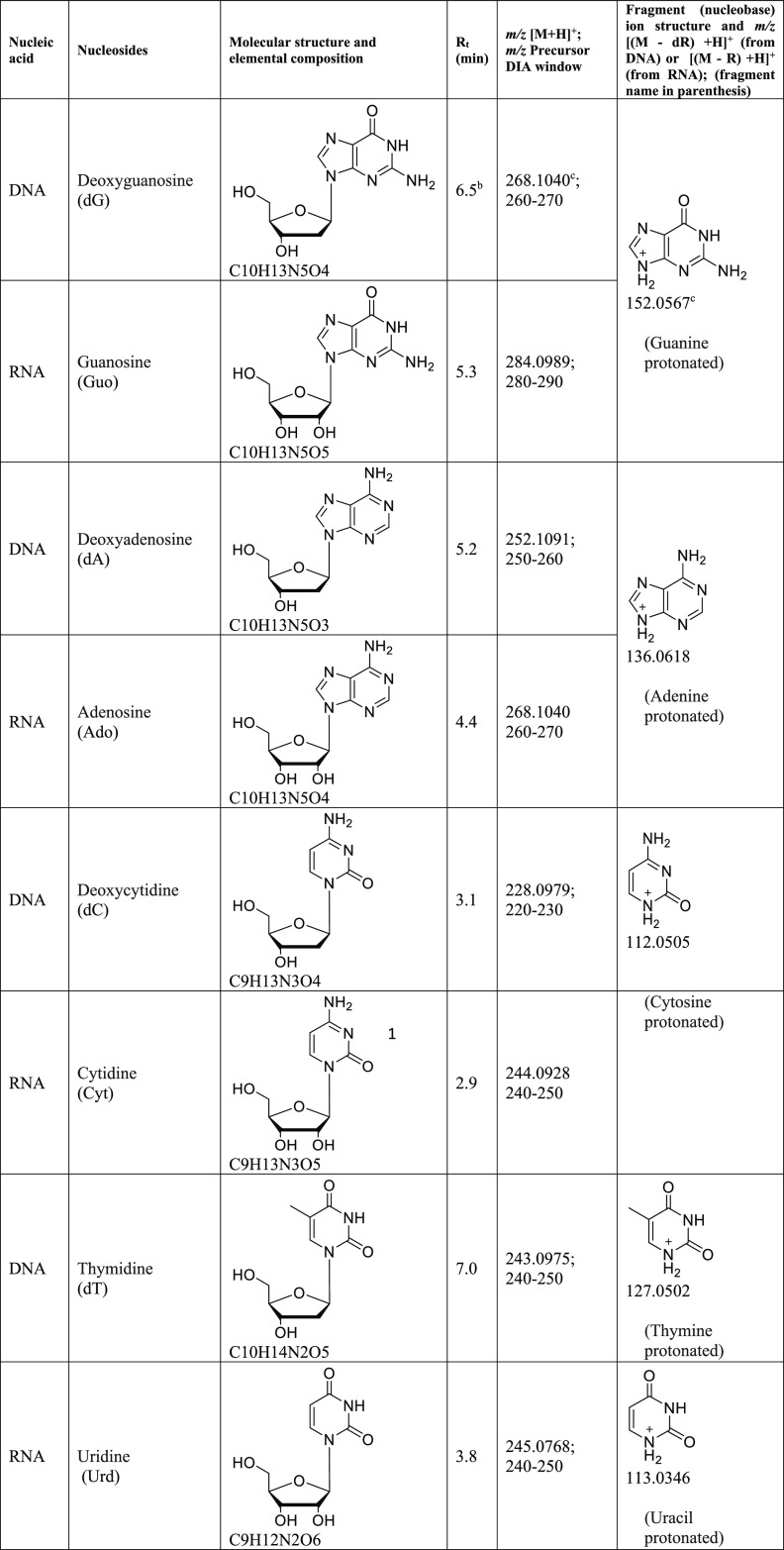
DNA and RNA Nucleosides and Structural Characteristics Used for Their Selective Identification^a^

a The features include calculated *m*/*z* of the molecular ion as [M + H]^+^ and that of the corresponding nucleobase fragment ion formed from the neutral loss of the deoxyribose (dR in DNA) or ribose (R in RNA) moiety, respective DIA window where the specific fragment ions were detected and chromatographic retention.

b Retention time (*R*_t_) observed using the employed LC conditions.

c *m*/*z* of the molecular and fragment ions are calculated using ChemDraw Professional 16.

**Figure 2:**
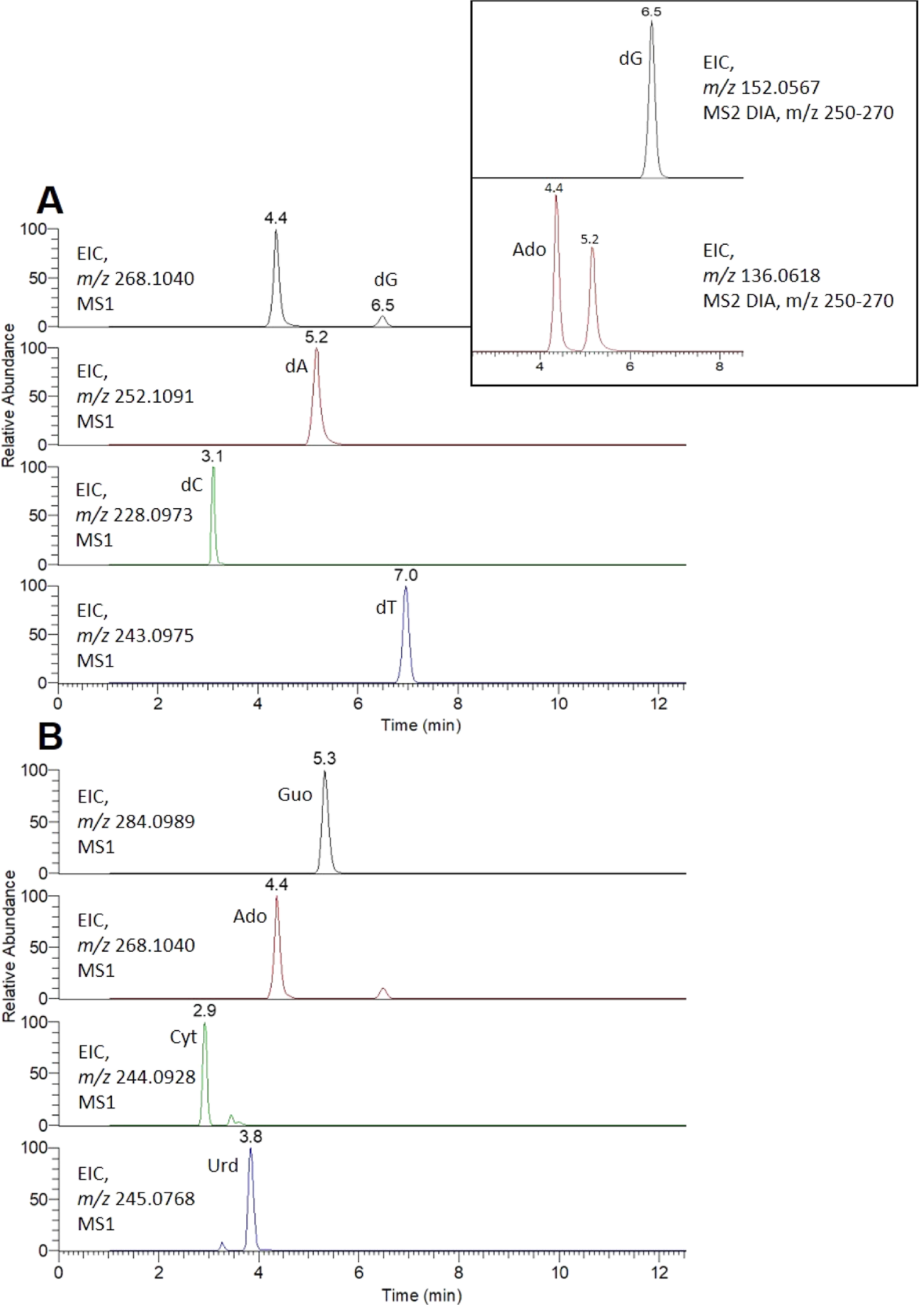
EIC using *m*/*z* [M + H]^+^ (± 5 ppm) of each 2′-deoxyribonucleosides (A) and ribonucleosides (B). In (A), panel 1 for *m*/*z* 268.1040, corresponding to the molecular ion for dG, showed two peaks. Inset shows EIC of nucleobase fragments, guanine (*m*/*z* 152.0567) and adenine (*m*/*z* 136.0618) from the same DIA window range of *m*/*z* 250–270, which demonstrate that the peak at 6.5 min in panel 1 corresponds to dG, whereas the peak at 4.4 min in the same panel corresponds to Ado (also shown in B, panel 2). In both (A,B), all peak assignments were confirmed by comparison with respective standards.

As the exact mass of dG and Ado is the same, *m*/*z* 268.1040, two peaks were detected for the EIC in MS1 (Figure 2A). As seen in Table 1, dG and Ado give the nucleobase guanine ion (fragment *m*/*z* 152.0567) and adenine ion (*m*/*z* 136.0618), respectively. Using this difference in MS2 and searching for the respective fragment ions (guanine and adenine) in MS2 under an experiment with the DIA window range *m*/*z* 250–270, dG was shown to elute at 6.5 min (inset in Figure 2A). In the MS2 insert for *m*/*z* 136.0618 (adenine ion), two peaks were detected, one for Ado (4.4 min) and the other for dA (5.2 min). Both peaks were detected because their respective precursors (Ado: *m*/*z* 268.1040; dA: *m*/*z* 252.1091) are in the same DIA window range, *m*/*z* 250–270. On the other hand, in the MS2 insert for *m*/*z* 152.0567 (guanine ion), only dG and not Guo was detected because the precursor for Guo (*m*/*z* 284.0989) was outside the selected DIA window range. All four nucleosides of DNA and the four nucleosides of RNA were detected in the amphipod samples and characterized using our method (Table 1, Figure 2).

### 3.2 RNA Adduct Detection

The RNA adducts were screened using targeted- and untargeted approaches. In the targeted approach, we used the database MODOMICS, which has 163 known endogenous RNA adducts (Table S1). Using TraceFinder, these adducts were searched for in the MS raw files based on high mass accuracy in MS1 spectra and the presence of the ribose fragment ion, *m*/*z* 133.04954 ± 5 ppm, within the same chromatographic retention in the corresponding MS2 DIA window (for adducts within the employed mass range *m*/*z* 200–350). As a result, nine putative RNA adducts were detected with the observed *m*/*z* [M + H]^+^ accuracy of the respective ribonucleoside adducts within 5 ppm (Table 2). In addition, HRAM measurements allowed a preliminary confirmation of the elemental composition of the detected ribonucleoside adducts (identification described in Section 3.3).

**Table 2:**
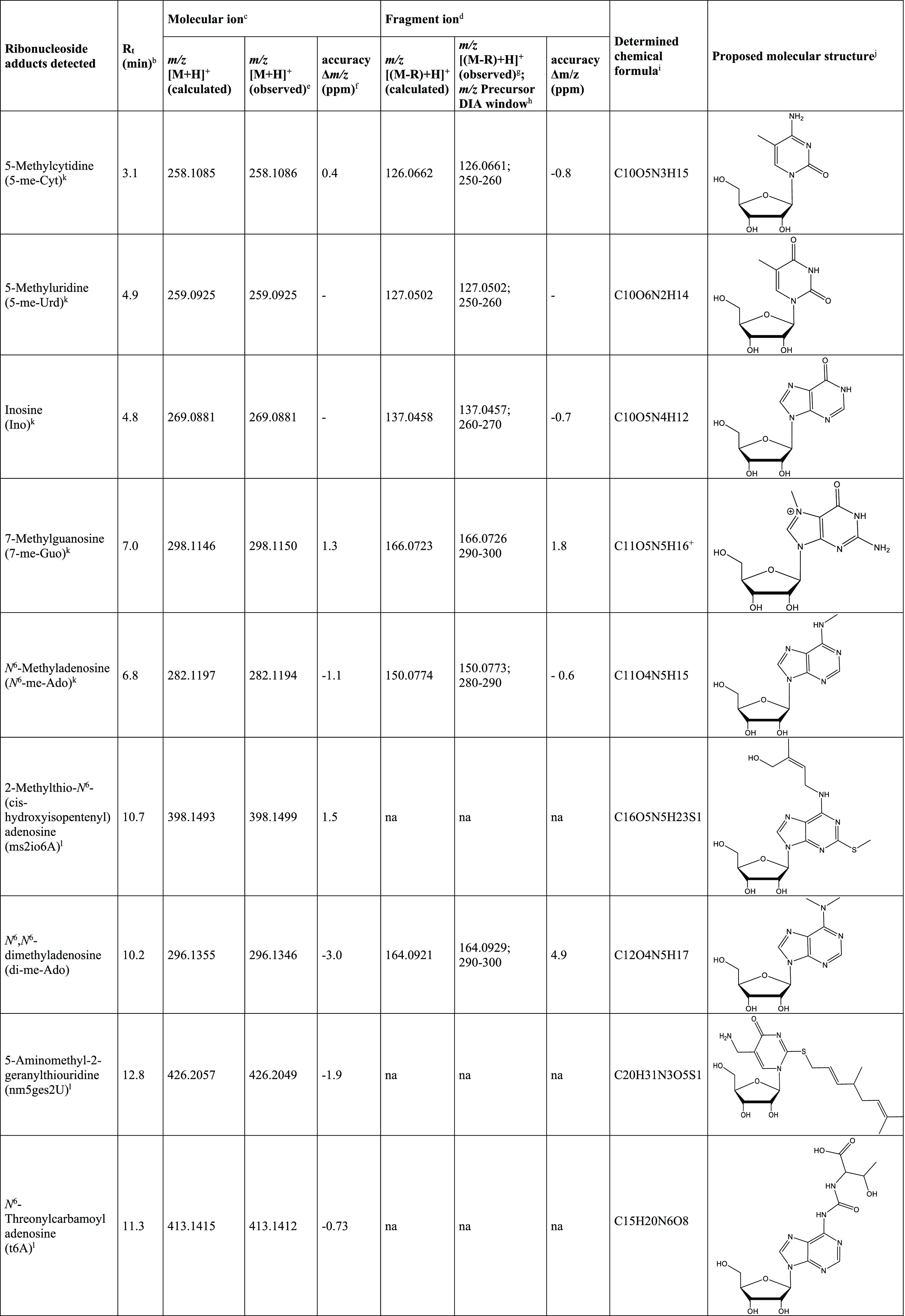
RNA Adducts Characterized in the Amphipod Samples Based on Retention Time and High Mass Accuracy Data^a^

a The structural characterization features, including chemical formula, accurate mass data of the ribonucleoside adducts as the molecular ion and nucleobase adduct (fragment) ion, and chromatographic retention time, are shown for each adduct.

b Retention time (*R*_t_) observed using the employed LC conditions.

c Molecular ion of the RNA adduct measured as the protonated ribonucleoside adduct ion.

d Fragment ion, from loss of the ribose moiety (R) on the molecular ion, measured as the protonated nucleobase adduct ion.

e *m*/*z* [M + H]^+^ corresponding to the protonated ribonucleoside adduct detected in FS spectra.

f Accuracy in ppm calculated as the difference between the theoretical and observed masses.

g *m*/*z* [(M-R)+H]^+^ corresponding to the protonated nucleobase adduct detected in the MS2 spectra of the selected DIA window.

h DIA window range in *m*/*z* based on the inclusion of the molecular ion and where the fragment ions were detected in the corresponding MS2 spectra.

i Elemental composition of the ribonucleoside adduct obtained from MODOMICS and determined in the amphipods based on the HRAM data of the molecular ion.

j Structure of the ribonucleoside adduct obtained from MODOMICS and proposed in the amphipods based on the HRAM data of molecular and fragment ions and comparison with the reference standard when available.

k Retention time and HRAM data of molecular and fragment ions compared with the reference standard.

l Compound not found in *nLossFinder* because it is out of the detection range up to *m*/*z* 350. na; data not available on fragmentation as the molecular ion is outside the measured DIA window range of *m*/*z* 200–350.

The untargeted screening approach used here considered that the fragmentation by MS/MS of ionized ribonucleoside adducts (on Ado, Guo, Cyt, and Urd), [M + H]^+^, occurs with the neutral loss of the ribose (R) moiety, which is a common characteristic of all RNA, resulting in a corresponding specific nucleobase adduct fragment ion [(M – R)+H]^+^. The search for ribonucleoside adducts with *nLossFinder* using the ribose neutral loss (R, 132.0423 Da), resulting from the difference between the precursor ion in MS1 and its corresponding specific fragment ion in the MS2 relevant DIA window, yielded both the precursor and fragment ions present within the same chromatographic retention time.

**Figure 3:**
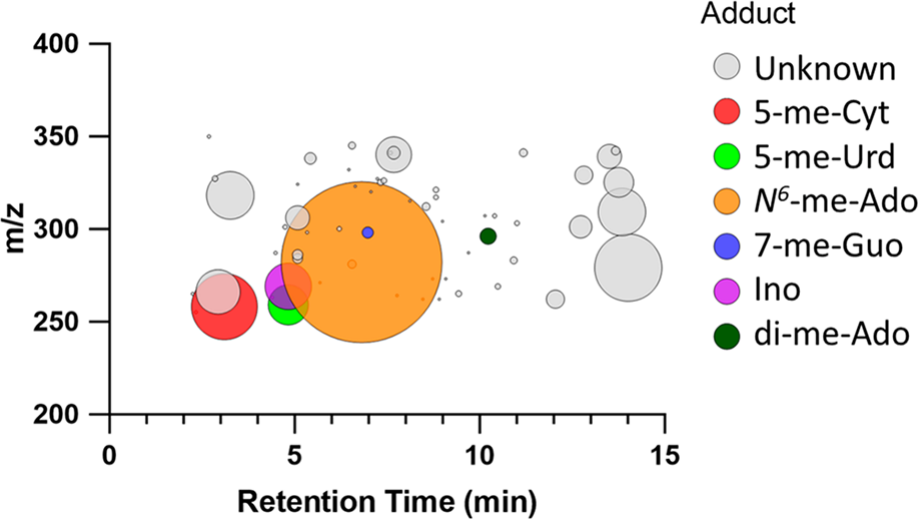
RNA adductome map based on the detected adducts in amphipods. Each circle represents an individual ribonucleoside adduct, with retention time plotted on the *X*-axis and *m*/*z* of the precursor ion on the *Y*-axis, and the circle size corresponding to relative abundance of each adduct (based on the EIC peak area of the adduct and normalized to that of Guo, adduct area × 10^2^/Guo area). All structurally identified adducts are shown in respective colors.

We applied the untargeted screening approach for RNA adductomics using *nLossFinder*, within the mass range *m*/*z* 200–350 of the nucleosides, based on the neutral loss of the ribose moiety (132.0423 Da ± 5 ppm). *nLossFinder* was developed and evaluated for the nontargeted detection of 2′-deoxyribonucleoside adducts using the corresponding deoxyribose (dR) as neutral loss,^22^ and here, we adapted it for RNA adductomics. In total, 60 putative RNA adducts were detected in the amphipods (Table S2, Figure 3). Six of the nine adducts detected by comparing with the MODOMICS library were found in the *nLossFinder* output (Table S2). The *m*/*z* of the protonated ribonucleoside adducts for the three RNA adducts not detected by *nLossFinder* were 398.1493 (ms2io6A), 426.2057 (nm5ges2U), and 413.1415 (t6A). All these adducts were beyond *m*/*z* 350, explaining that they were not detectable by *nLossFinder* with the DIA window set to max *m*/*z* 350. However, all adducts in the range *m*/*z* 200—350 detected using the targeted approach were detected by *nLossFinder*, which, at least, partly validates the use of this software for the nontargeted detection of RNA adducts. No nucleosides were detected in the blank samples prepared by the same procedure but in the absence of DNA/RNA.

### 3.3 Structural Identification of RNA Adducts

All 60 RNA adducts detected in the amphipods were characterized (Table S2) based on their respective observed accurate mass of the ribonucleoside adduct ion and its corresponding nucleobase adduct ion and the retention time under the employed LC conditions. For the six adducts found in the MODOMICS library (Table S2), elemental composition and chemical structures were proposed (Table 2). The mass difference between the observed *m*/*z* and calculated *m*/*z* of the ribonucleoside adduct molecular ion was from −3.0 to 1.5 ppm and that of the nucleobase adduct fragment ion was −0.8 to 4.9 ppm (Table 2).

**Figure 4:**
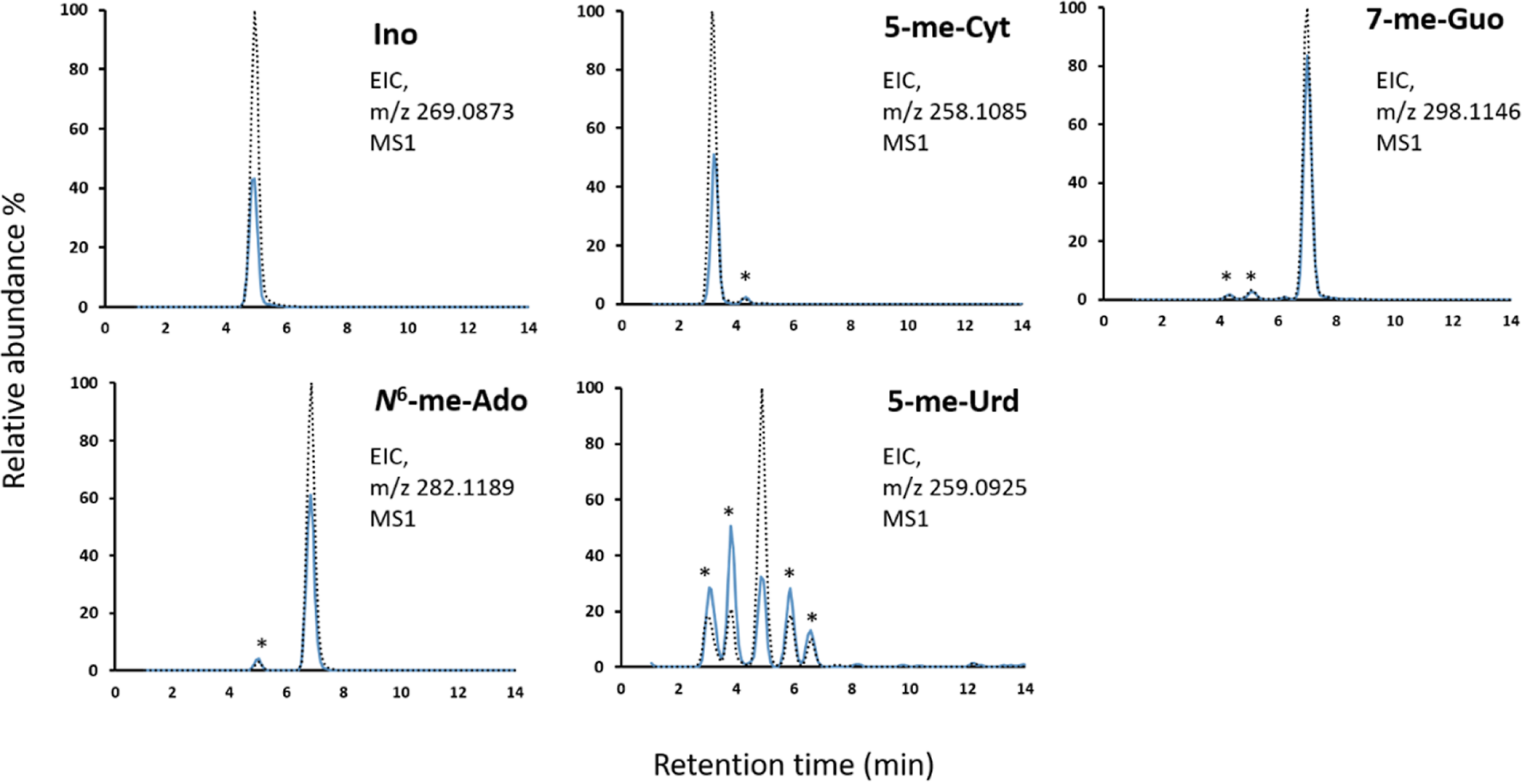
Identification of Ino, 5-me-Cyt, 7-me-Guo, *N*^*6*^-me-Ado, and 5-me-Urd in the amphipod RNA using respective standards. An overlap of the EIC peaks, using *m*/*z* of respective [M + H]^+^ (±5 ppm), corresponding to the ribonucleoside adducts in amphipod samples before (solid line) and after (dotted line) spiking with the respective standards to confirm the identification. EIC for *m*/*z* 259.0925 in amphipod showed several peaks, but only the third peak (retention time 4.9 min) had a relative increase in intensity after spiking the standard, indicating that this peak corresponds to 5-me-Urd. The peaks marked with an asterisk (*) are not affected by the spiking, suggesting that they might be potential isomers in EIC for 5me-Urd (which also had the fragment nucleobase adduct ion detected) or minor impurities in EICs for 5-me-Cyt, *N*^*6*^-me-Ado, and 7-me-Guo (for which the fragment corresponding to the respective nucleobase adduct ions was not detected).

Structures of five adducts, Ino, 5-me-Cyt, 7-me-Guo, *N*^*6*^-me-Ado, and 5-me-Urd, were confirmed by comparison of LC-elution and HRAM data with the respective reference standards. When the digested samples were spiked with the standard solutions, an overlay of the EIC peaks showed a total retention time matching the increased relative intensity at that particular retention time (Figure 4). In the amphipods, the EIC of *m*/*z* 259.0925 corresponding to the molecular ion for 5-me-Urd showed five peaks (at retention times 3.0, 3.8, 4.9, 5.9, and 6.5 min). After spiking with the reference standard 5-me-Urd, the peak at 4.9 min relatively increased in intensity, implying this being the 5-me-Urd adduct. Further, the corresponding nucleobase adduct fragment ion, *m*/*z* 127.052, was observed under each of the five chromatographic peaks in the correct DIA window of *m*/*z* 250–260 (Table 2) as that window (*m*/*z* 250–260) would constitute the precursor *m*/*z* 259.0925 of 5-me-Urd. This finding suggests that the peaks other than at 4.9 min are possible structural isomers of 5-me-Urd, which could be the methylation on positions other than C-5 on Urd. EICs for 5-me-Cyt, *N*^*6*^-me-Ado, and 7-me-Guo showed minor peaks (below 5% of the identified adduct and marked with an asterisk in Figure 4) in addition to the identified adduct, with precursor mass matching within 5 ppm, but the corresponding fragment ion mass was not detected, suggesting these to be trace impurities.

## 4 Discussion

A novelty of our work is to provide a workflow for simultaneous RNA and DNA adductomics analysis in the same sample. Moreover, an adductome profiling analysis of the pooled amphipod sample was performed using the newly developed method as a proof-of-principle study. The extraction method involved heating the homogenized amphipod bodies in the presence of an aqueous Chelex ion-exchange resin suspension. This extraction method is advantageous for the simultaneous extraction of the nucleic acids for adductome analysis as it yields both DNA and RNA and is relatively simple compared to, for instance, organic extraction using phenol-chloroform and solid-phase extraction methods. The cells lyse, and the polar biochemical compounds bind to the resin beads,^31^ whereas the nucleic acids remain in the water phase. DNA is hydrolyzed to 2′-deoxyribonucleosides by a standard enzymatic digestion procedure. RNA is prone to hydrolysis;^32^ thus, most of it is expected to be cleaved to the ribonucleosides already at the extraction step, which includes heating up to 65 °C. Thus, the RNA extracted by this method is highly digested and would not be suitable for sequencing or PCR-based techniques; however, this does not preclude using this RNA for the analysis of the ribonucleosides with or without adducts.

**Figure 5:**
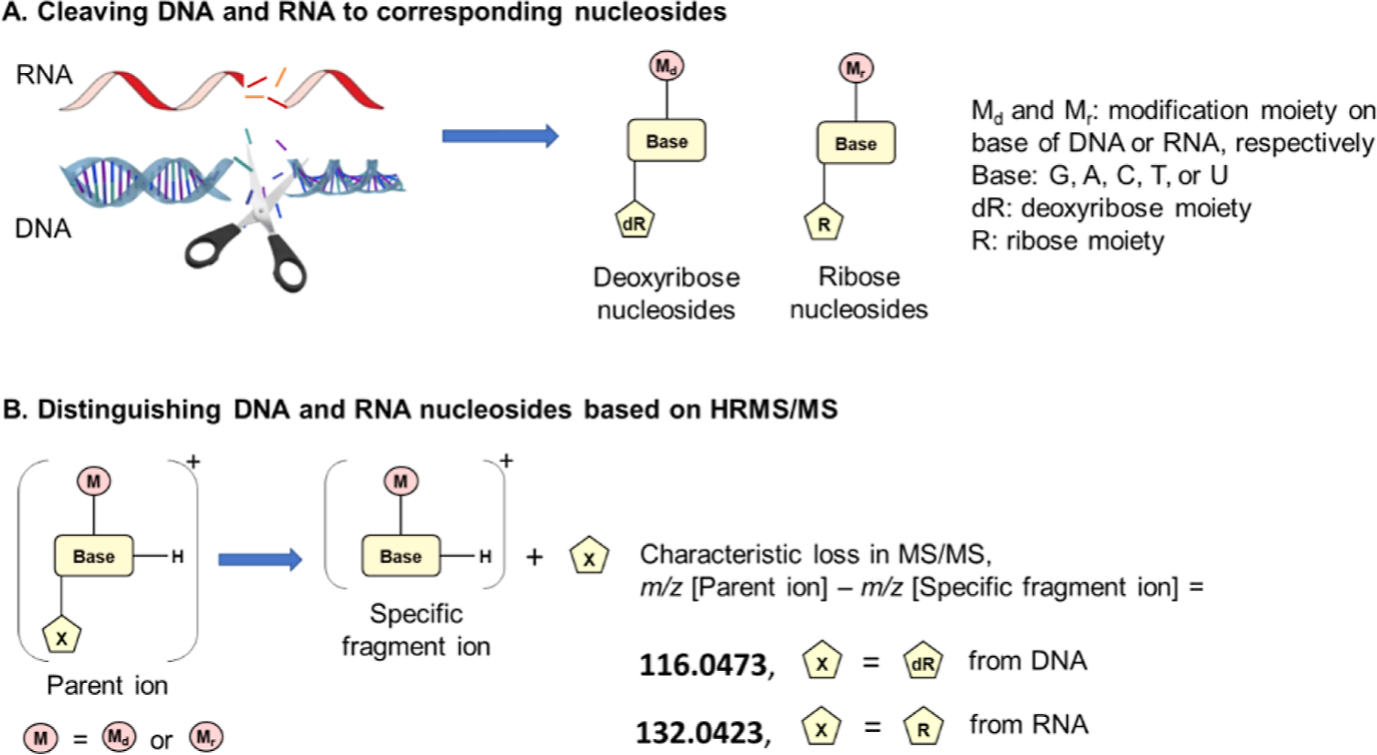
Representation of nucleic acid cleavage (A) and selective detection (B) of RNA- and DNA-nucleoside adducts. After hydrolysis of DNA and RNA, the nucleosides are characterized by three parts: the base, the modifier part, and the sugar ring deoxyribose (dR from DNA) or ribose (R from RNA). Detection, using HRMS, is based on the identification of the two ions—parent (molecular) ion and specific fragment (nucleobase adduct) ion—and the neutral loss that differs between DNA (dR, 116.0473 Da) and RNA (R, 132.0423 Da).

The resulting cleaved mixture consisted of ribonucleosides and 2′-deoxyribonucleosides (Figure 5A), unmodified and modified. LC-HRMS was used for the characterization of the protonated unmodified nucleosides (Table 1) from DNA (dG, dA, dC, and dT) and RNA (Guo, Ado, Cyt, and Urd). In the proposed approach, the DNA adducts and RNA adducts resulting in modified 2′-deoxyribonucleosides and modified ribonucleosides, respectively, were selectively analyzed using the same LC-HRMS runs, employing the specific difference in their neutral loss upon MS-fragmentation. This fragmentation of the respective protonated DNA and RNA nucleoside adducts yields characteristic neutral losses: 116.0473 Da, which is the specific mass for the deoxyribose (dR) moiety, and 132.0423 Da, the specific mass for the ribose (R) moiety (Figure 5B). For known RNA and DNA adducts, e.g., those available from a library or previous work, the calculated accurate *m*/*z* of the corresponding protonated nucleoside adducts can be used to extract ion chromatograms from MS1, and selectivity is evaluated by the specific fragment ion *m*/*z* in MS2. For the nontargeted detection of RNA and DNA adducts, the characteristic neutral loss, 116.473 Da specific for DNA adducts and 132.0423 Da specific for RNA adducts, can be used for selective screening of the respective nucleoside adducts.

In the pooled mixture of nucleosides from the amphipod samples, nearly 150 putative DNA adducts were detected by employing *nLossFinder* programmed for the nontargeted detection of DNA adducts using the neutral loss 116.0473 Da (Sousa et al. 2021, also shows the DNA adductome map for amphipods).^22^ Among them were the epigenetic markers, 5-me-dC and *N*^*6*^-me-dA, and the oxidative stress marker, 8-oxo-dG. See Sousa et al. 2021^22^ for a detailed description of the approach for DNA adductomics and a complete list of the identified DNA adducts.

In addition to these DNA adducts, while reanalyzing the HRMS-raw files, 60 putative RNA adducts were detected in the amphipods, and 9 were structurally identified based on high mass accuracy (Table 2). However, several known RNA modifications, listed in the MODOMICS database, were not found in the amphipod samples, possibly because of their low abundance or poor ionization efficiency. The key advantage of the LC–MS methodology is the highly quantitative nature of the approach. We obtained EICs for each adduct using the HRAM data in MS1 and assuming an equal MS response, with the resulting peak areas providing relative abundances of the adducts and visualized as an adductome map (Figure 3). Normalizing each nucleoside adduct to dG (for DNA adducts) and to Guo (for RNA adducts) not only takes into account any differences in the hydrolysis of the nucleic acids but also avoids the use of expensive isotope-labeled standards. A considerably major RNA modification in the amphipods, sentinel species, used to assess the health status of the Baltic Sea, based on the MS1 peak area was identified as a deamination product, inosine (Ino in Figure 3, MS1 *m*/*z* 269.0873). This finding is supported by the fact that inosine is one of the most abundant post-transcriptional RNA modifications.^33^ Moreover, adenosine to inosine conversion is catalyzed by the adenosine deaminase action on RNA enzyme, and the aberrations of the enzyme’s activity have been associated with, e.g., metabolic disorders and cancer,^34−37^ indicating the potential use of inosine as a biomarker in (eco)toxicological studies.

Screening for unknown RNA adducts is less studied than screening for DNA adducts. Recently, Takeshita and Kanaly (2019)^15^ used the detection of RNA adducts in benzo[*a*]pyrene-exposed human hepatoma cell line to confirm putative DNA adducts detected by MS-based adductomics. DNA and RNA were extracted separately using different extraction kits, followed by digestion. Benzo[*a*]pyrene-diolepoxide nucleoside adducts from both DNA and RNA were detected using HRMS and manual monitoring of the neutral loss, deoxyribose for protonated 2′-deoxynucleoside adducts and ribose for protonated ribonucleoside adducts.

The RNA modifications detected here and those reported in the literature^38−40^ mainly involve small chemical groups added to any of the four ribonucleosides. All the adducts identified in our study (Table 2) are known to be constituents of the epitransciptome^41,42^ comprising post-transcriptional alterations that do not alter the RNA sequence.^43,44^ Our MS-based approach does not allow us to differentiate as to which RNA type these modifications were adducted. However, the literature suggests that most high-variety modifications, such as 5-me-Cyt, 5-me-Urd, Ino, *N^6^*-me-Ado, di-me-Ado, ms2io6A, and t6A, are on tRNA and rRNA,^45−47^ whereas simple methylations, e.g., 5-me-Cyt and *N^6^*-me-Ado, are usually associated with mRNA.^37,48−51^ These epitranscriptomic RNA modifications can affect various translation processes and RNA stability, consequently impacting diverse cellular and biological pathways, including the tightly regulated gene expression.^43,45,52,53^

To our knowledge, this is the first study on nontargeted detection of modified ribonucleosides over the mass range of *m*/*z* 200–350 in an environmental context using a sentinel species as a test organism. Of the 60 RNA adducts detected by the nontargeted approach, 54 RNA modifications have not been structurally identified, and none of the masses for these unidentified modifications correspond to the modifications in MODOMICS. To facilitate further efforts on the characterization and identification of these unknown RNA adducts, we provided the following information: (i) molecular ion (*m*/*z*) of the protonated ribonucleoside adduct from MS1 with high mass accuracy; (ii) retention time under the employed LC conditions providing relative hydrophilicity; (iii) MS-fragmentation pattern including *m*/*z* of the nucleobase adduct fragment ion, obtained after neutral loss of the ribose moiety, with high mass accuracy; and (iv) relative abundance based on MS1 peak areas assuming equal MS response. There is scope for improving the detection limit, e.g., by employing nano-LC;^54^ apart from their cost and maintenance requirement; a disadvantage with nano-LC is the requirement of longer chromatographic runs. Further, as the possibility of false positives, including isotopes and ESI adducts, assigned as individual adducts cannot be ruled out, future work is required toward structural identification of these adducts to aid in characterization of the RNA adductome. Improving the sensitivity for adductome analysis along with knowing the chemical structure of the modifications and the extent of their dysregulation by various environmental stressors will facilitate the development of adductome-based biomarkers and their use for environmental monitoring of biological effects.

Using the multi-adductomics methodology developed in the current work, there are still many adducts that have not been structurally identified in the amphipods. A main reason for this is that we used a neutral loss approach combined with high mass accuracy for adductomics and not relied only on the comparison with a library (such as MODOMICS). This, in fact, is a significant aspect of our work as we have not only performed adduct screening based on earlier known adducts but also have developed a state-of-the-art untargeted approach that allows us to detect earlier unknown adducts. Further work is required to structurally identify the putative novel adducts detected in the amphipods and use experimental studies to identify potential association of chemical exposure with these nucleic acid modifications.

## 5 Conclusions

We introduce an approach for simultaneous RNA and DNA adductomics using a single injection on LC-Orbitrap HRMS instrumentation. RNA and DNA adducts can be detected by measuring modified ribonucleosides and 2′-deoxyribonucleosides, respectively. The workflow for multi-adductomics was demonstrated by analyzing amphipod RNA and DNA adducts as a proof-of-principle study. Our MS approach with FS provided HRAM data in the MS1, and DIA resulted in comprehensive fragmentation, providing MS2 data for all ions across a broad selected *m*/*z* range sequentially. In the MS analysis, a challenge of the same *m*/*z* for the protonated dG and Ado was overcome (i) by using MS2 fragment data of the specific nucleobases, G and A, which have different masses and (ii) by the difference in their relative chromatographic retention. For the DNA adductome, *nLossFinder* was employed to detect nucleoside adducts by tracking a neutral loss under MS fragmentation, with the structural identification primarily based on literature knowledge of possible modifications on the nucleobases. For the RNA adductome, *nLossFinder* screening was combined with the MODOMICS database of RNA modifications, which aided the structural identification of several adducts. In the amphipods, we structurally identified simple methylated adducts on DNA (5-me-dC and *N*^*6*^-me-dA) and RNA (5-me-Cyt, *N*^*6*^-me-Ado, 5-me-rU, and 7-me-Guo), genotoxic adducts (8-oxo-dG), and more complex post-transcriptional modifications (di-me-Ado, ms2io6A, m5ges2U, and t6A). Information on *m*/*z* of molecular and fragment ions related to the chemical formula and chromatographic retention related to hydrophilicity was provided for 54 RNA modifications detected in the amphipods. More work, including MS3 experiments and exposure studies with model organism, is needed to facilitate structural characterization and validation of these adducts. The discovery of new RNA modifications opens new venues in analytical validation and understanding their biological significance on epitranscriptome and the impact of contaminants on the adductome, which can support future applications of specific adducts as molecular biomarkers in environmental monitoring.

## Supporting Information Available

Identification of ribonucleosides in the amphipods; RNA adduct database extracted from MODOMICS; and *nLossFinder* output list of putative RNA adducts in amphipods (PDF)
