# Supplementary material for: Simultaneous RNA and DNA Adductomics Using Single Data-Independent Acquisition Mass Spectrometry Analysis
Source: Chem Res Toxicol. 2023 Aug 11;36(9):1471–82. doi: 10.1021/acs.chemrestox.3c00041 (PMC10523582; doi:10.1021/acs.chemrestox.3c00041)
Supplement: Supplementary file 1 — tx3c00041_si_001.pdf [file tx3c00041_si_001.pdf]

Supplementary Material

Simultaneous RNA and DNA adductomics using single data-independent acquisition mass spectrometry analysis

Giulia Martella[1,], Nisha H. Motwani[2], Zareen Khan[1], Pedro F.M. Sousa[3], Elena Gorokhova[1] and Hitesh V. Motwani[1,*]

[1]Department of Environmental Science, Stockholm University, SE-106 91 Stockholm, Sweden

[2]School of Natural Sciences, Technology and Environmental Studies, Södertörn University, SE-14189 Huddinge, Sweden

[3]Department of Materials and Environmental Chemistry, Stockholm University, SE-106 91 Stockholm, Sweden

[*]Corresponding author: hitesh.motwani@aces.su.se

Contents

Figure S1. Identification of adenosine, cytidine, guanosine and uridine in the amphipods using respective standards. An overlap of EIC peaks using *m/z* of respective molecular ions, $[M+H]^+$, corresponding to the ribonucleosides in amphipod samples before (blue solid line) and after (black dotted line) spiking of the respective standards confirmed the identification. *Page S2*

Table S1. List of RNA adducts showing Name, Short Name, Formula, Monoisotopic mass and Protonated mass, obtained from MODOMICS database. The protonated mass, *m/z* $[M+H]^+$, were screened for in the MS raw files from the amphipod samples using TraceFinder. Nine adducts were detected in the amphipods, which are highlighted in bold. *Page S3*

Table S2. List of the 60 putative RNA adducts detected in the amphipods using *nLossFinder*. The table includes the *m/z* observed of the precursor ions in MS1 (MS1_mz) and of corresponding nucleobase adduct fragment ions in MS2 (MS2_mz), difference between the MS1 and MS2 ions (mz_diff, i.e., observed neutral loss), retention times in min (MS1_time, MS2_time), absolute intensities (MS1_int, MS2_int) and peak areas (MS1_Area, MS2_Area) for the respective MS1 and MS2 ions. The DIA window central *m/z* (DIA_mz) and the neutral loss error (nLoss_err) are also shown. The latter is expressed as Δppm between the neutral loss calculated (132.0423) and that observed. Six of the adducts were structurally identified (shown in Table 2), which are highlighted in bold. *Page S11*

Figure S1. Identification of adenosine, cytidine, guanosine and uridine in the amphipods using respective standards. An overlap of EIC peaks using *m/z* of respective molecular ions, $[M+H]^+$, corresponding to the ribonucleosides in amphipod samples before (blue solid line) and after (black dotted line) spiking of the respective standards confirmed the identification.

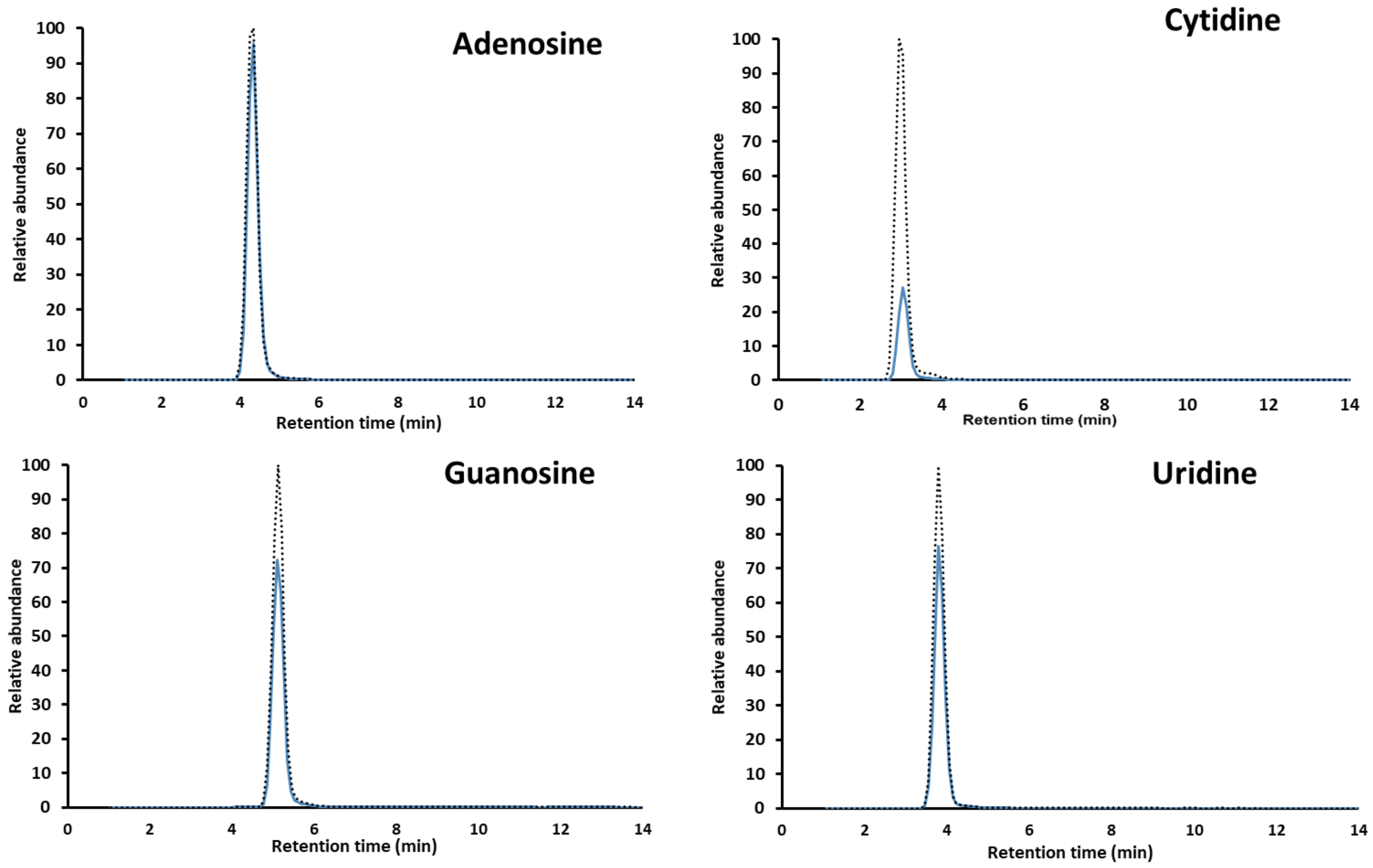

Table S1. List of RNA adducts showing Name, Short Name, Formula, Monoisotopic mass and Protonated mass, obtained from MODOMICS database. The protonated mass, *m/z* $[M+H]^+$, were screened for in the MS raw files from the amphipod samples using TraceFinder. Nine adducts were detected in the amphipods, which are highlighted in bold.

Name	Short Name	Formula	Monoisotopic mass (Exact mass)	Protonated mass
1,2-O-dimethyladenosine	m1Am	C12O4N5H17	295.1281	296.1354
1,2-O-dimethylguanosine	m1Gm	C12O5N5H17	311.123	312.1303
1,2-O-dimethylinosine	m1Im	C12O5N4H16	296.1121	297.1194
1-methyl-3-(3-amino-3-carboxypropyl)pseudouridine	m1acp3Y	C14O8N3H21	359.1329	360.1402
1-methyladenosine	m1A	C11O4N5H15	281.1124	282.1197
1-methylguanosine	m1G	C11O5N5H15	297.1073	298.1146
1-methylinosine	m1I	C11O5N4H14	282.0964	283.1037
1-methylpseudouridine	m1Y	C10O6N2H14	258.0852	259.0925
2,8-dimethyladenosine	m2,8A	C12H17N5O4	295.1281	296.1354
2- methylthiomethylenethio-N6-isopentenyl-adenosine	msms2i6A	C17H25N5O4S2	427.1347	428.1420
2-geranylthiouridine	ges2U	C19H28N2O5S	396.1719	397.1792
2-lysidine	k2C	C15O6N5H25	371.1805	372.1878
2-methyladenosine	m2A	C11O4N5H15	281.1124	282.1197
2-methylthio cyclic N6-threonylcarbamoyladenosine	ms2ct6A	C17H21N5O7S	439.1162	440.1235
**2-methylthio-N6-(cis-hydroxyisopentenyl) adenosine**	**ms2io6A**	**C16O5N5H23S1**	**397.1420**	**398.1493**
2-methylthio-N6-hydroxynorvalylcarbamoyladenosine	ms2hn6A	C17O8N6H24S1	472.1376	473.1449
2-methylthio-N6-isopentenyladenosine	ms2i6A	C16O4N5H23S1	381.1471	382.1544

2-methylthio-N6-methyladenosine	ms2m6A	C12O4N5H17S1	327.1001	328.1074
2-methylthio-N6-threonylcarbamoyladenosine	ms2t6A	C16O8N6H22S1	458.1220	459.1293
2-selenouridine	se2U	C9O5N2H12Se1	305.9919	306.9992
2-thio-2-O-methyluridine	s2Um	C10O5N2H14S1	274.0623	275.0696
2-thiocytidine	s2C	C9O4N3H13S1	259.0627	260.0700
2-thiouridine	s2U	C9O5N2H12S1	260.0467	261.0540
2-O-methyladenosine	Am	C11O4N5H15	281.1124	282.1197
2-O-methylcytidine	Cm	C10O5N3H15	257.1012	258.1085
2-O-methylguanosine	Gm	C11O5N5H15	297.1073	298.1146
2-O-methylinosine	Im	C11O5N4H14	282.0964	283.1037
2-O-methylpseudouridine	Ym	C10O6N2H14	258.0852	259.0925
2-O-methyluridine	Um	C10O6N2H14	258.0852	259.0925
2-O-methyluridine 5-oxyacetic acid methyl ester	mcmo5Um	C13H18N2O9	346.1012	347.1085
2-O-ribosyladenosine (phosphate)	Ar(p)	C15O11N5H22P1	479.1053	480.1126
2-O-ribosylguanosine (phosphate)	Gr(p)	C15O12N5H22P1	495.1003	496.1076
2-O-Methyl-5-hydroxymethylcytidine	hm5Cm	C11H17N3O6	287.1117	288.1190
3,2-O-dimethyluridine	m3Um	C11O6N2H16	272.1008	273.1081
3-(3-amino-3-carboxypropyl)-5,6-dihydrouridine	acp3D	C13H21N3O8	347.1329	348.1402
3-(3-amino-3-carboxypropyl)pseudouridine	acp3Y	C13H19N3O8	345.1172	346.1245
3-(3-amino-3-carboxypropyl)uridine	acp3U	C13O8N3H19	345.1172	346.1245
3-methylcytidine	m3C	C10O5N3H15	257.1012	258.1085

3-methylpseudouridine	m3Y	C10O6N2H14	258.0852	259.0925
3-methyluridine	m3U	C10O6N2H14	258.0852	259.0925
4-demethylwyosine	imG-14	C13O5N5H15	321.1073	322.1146
4-thiouridine	s4U	C9O5N2H12S1	260.0467	261.0540
5,2-O-dimethylcytidine	m5Cm	C11O5N3H17	271.1168	272.1241
5,2-O-dimethyluridine	m5Um	C11O6N2H16	272.1008	273.1081
5-(carboxyhydroxymethyl)-2â€²-O-methyluridine methyl ester	mchm5Um	C13H18N2O9	346.1012	347.1085
5-(carboxyhydroxymethyl)uridine methyl ester	mchm5U	C12O9N2H16	332.0856	333.0929
5-(isopentenylaminomethyl)-2-thiouridine	inm5s2U	C15H23N3O5S	357.1358	358.1431
5-(isopentenylaminomethyl)-2â€²-O-methyluridine	inm5Um	C16H25N3O6	355.1743	356.1816
5-(isopentenylaminomethyl)uridine	inm5U	C15H23N3O6	341.1587	342.1660
**5-aminomethyl-2-geranylthiouridine**	**nm5ges2U**	**C20H31N3O5S**	**425.1984**	**426.2057**
5-aminomethyl-2-selenouridine	nm5se2U	C10O5N3H15Se1	337.0176	338.0249
5-aminomethyl-2-thiouridine	nm5s2U	C10O5N3H15S1	289.0732	290.0805
5-aminomethyluridine	nm5U	C10O6N3H15	273.0961	274.1034
5-carbamoylhydroxymethyluridine	nchm5U	C11H15N3O8	317.0859	318.0932
5-carbamoylmethyl-2-thiouridine	ncm5s2U	C11H15N3O6S	317.0681	318.0754
5-carbamoylmethyl-2-O-methyluridine	ncm5Um	C12O7N3H17	315.1066	316.1139
5-carbamoylmethyluridine	ncm5U	C11O7N3H15	301.0910	302.0983
5-carboxyhydroxymethyluridine	chm5U	C11O9N2H14	318.0699	319.0772

5-carboxymethyl-2-thiouridine	cm5s2U	C11O7N2H14S1	318.0522	319.0595
5-carboxymethylaminomethyl-2-geranylthiouridine	cmnm5ges2U	C22H33N3O7S	483.2039	484.2112
5-carboxymethylaminomethyl-2-selenouridine	cmnm5se2U	C12O7N3H17Se1	393.0239	394.0312
5-carboxymethylaminomethyl-2-thiouridine	cmnm5s2U	C12O7N3H17S1	347.0787	348.0860
5-carboxymethylaminomethyl-2â€²-O-methyluridine	cmnm5Um	C13O8N3H19	345.1172	346.1245
5-carboxymethylaminomethyluridine	cmnm5U	C12O8N3H17	331.1016	332.1089
5-carboxymethyluridine	cm5U	C11O8N2H14	302.0750	303.0823
5-cyanomethyluridine	cnm5U	C11H13N3O6	283.0804	284.0877
5-formyl-2-O-methylcytidine	f5Cm	C11O6N3H15	285.0961	286.1034
5-formylcytidine	f5C	C10O6N3H13	271.0804	272.0877
5-hydroxycytidine	ho5C	C9H13N3O6	259.0804	260.0877
5-hydroxymethylcytidine	hm5C	C10O6N3H15	273.0961	274.1034
5-hydroxyuridine	ho5U	C9O7N2H12	260.0645	261.0718
5-methoxycarbonylmethyl-2-thiouridine	mcm5s2U	C12O7N2H16S1	332.0678	333.0751
5-methoxycarbonylmethyl-2â€²-O-methyluridine	mcm5Um	C13O8N2H18	330.1063	331.1136
5-methoxycarbonylmethyluridine	mcm5U	C12O8N2H16	316.0907	317.0980
5-methoxyuridine	mo5U	C10O7N2H14	274.0801	275.0874
5-methyl-2-thiouridine	m5s2U	C10O5N2H14S1	274.0623	275.0696
5-methylaminomethyl-2-geranylthiouridine	mnm5ges2U	C21H33N3O5S	439.2141	440.2214
5-methylaminomethyl-2-selenouridine	mnm5se2U	C11O5N3H17Se1	349.0341	350.0414
5-methylaminomethyl-2-thiouridine	mnm5s2U	C11O5N3H17S1	303.0889	304.0962

5-methylaminomethyluridine	mnm5U	C11O6N3H17	287.1117	288.1190
**5-methylcytidine**	**m5C**	**C10O5N3H15**	**257.1012**	**258.1085**
5-methyldihydrouridine	m5D	C10O6N2H16	260.1008	261.1081
**5-methyluridine**	**m5U**	**C10O6N2H14**	**258.0852**	**259.0925**
5-taurinomethyl-2-thiouridine	tm5s2U	C12O8N3H19S2	397.0614	398.0687
5-taurinomethyluridine	tm5U	C12O9N3H19S1	381.0842	382.0915
5(3-dephospho-CoA)	CoA(pN)	C21H32N7O13P2S	684.1254	685.1327
5(3-dephosphoacetyl-CoA)	acCoA(pN)	C22H40N7O15P2S	736.1778	737.1851
5(3 -dephosphomalonyl-CoA)	malonyl-CoA(pN)	C23H40N7O17P2S	780.1676	781.1749
5 (3 -dephosphosuccinyl-CoA)	succinyl-CoA(pN)	C24H41N7O17P2S	793.1755	794.1828
5 diphosphate end	p(pN)	PO3	78.9585	79.96578
5monophosphate end	(pN)	C5H7O10P2	288.9514	289.9587
5 nicotinamide adenine dinucleotide	NAD(pN)	C21H25N7O14P2	661.0935	662.1008
5 triphosphate end	pp(pN)	P3O9R	157.9170	158.9243
7-aminocarboxypropyl-demethylwyosine	yW-86	C17O7N6H22	422.1550	423.1623
7-aminocarboxypropylwyosine	yW-72	C18O7N6H24	436.1706	437.1779
7-aminocarboxypropylwyosine methyl ester	yW-58	C19O7N6H26	450.1863	451.1936
7-aminomethyl-7-deazaguanosine	preQ1tRNA	C12O5N5H17	311.1230	312.1303
7-cyano-7-deazaguanosine	preQ0tRNA	C12O5N5H13	307.0917	308.0990
**7-methylguanosine**	**m7G**	**C11O5N5H16+**	**298.1151**	**298.1151**
7-methylguanosine cap (cap 0)	m7Gpp(pN)	C11H15N5O11P2	455.0243	456.0316

8-methyladenosine	m8A	C11O4N5H15	281.1124	282.1197
adenosine	A	C10O4N5H13	267.0968	268.1041
agmatidine	C+	C14H25N7O4	355.1968	356.2041
alpha-dimethylmonophosphate cap	mm(pN)	C7H14O10P2	320.0062	321.0135
alpha-methylmonophosphate cap	m(pN)	C6H12O10P2	305.9906	306.9979
archaeosine	G+	C12H16N6O5	324.1182	325.1255
cyclic N6-threonylcarbamoyladenosine	ct6A	C15O7N6H18	394.1237	395.1310
cytidine	C	C9O5N3H13	243.0855	244.0928
dihydrouridine	D	C9O6N2H14	246.0852	247.0925
epoxyqueuosine	oQtRNA	C17O8N5H23	425.1547	426.1620
galactosyl-queuosine	galQtRNA	C23O12N5H33	571.2126	572.2199
gamma-methyltriphosphate cap	mpp(pN)	CH3O6P2	172.9405	173.9478
glutamyl-queuosine	gluQtRNA	C22O10N6H30	538.2023	539.2096
guanosine	G	C10O5N5H13	283.0917	284.0990
guanosine added to any nucleotide	pG(pN)	C10H13N5O8P	362.0502	363.0575
guanylylated 5â€² end (cap G)	Gpp(pN)	C10H12N5O14P3	440.0008	441.0081
hydroxy-N6-threonylcarbamoyladenosine	ht6A	C15H20N6O9	428.1292	429.1365
hydroxywybutosine	OHyW	C21O10N6H28	524.1867	525.1940
**inosine**	**I**	**C10O5N4H12**	**268.0808**	**269.0881**
isowyosine	imG2	C14O5N5H17	335.1230	336.1303
mannosyl-queuosine	manQtRNA	C23O12N5H33	571.2126	572.2199

methylated undermodified hydroxywybutosine	OHyWy	C19H26N6O8	466.1812	467.1885
methylwyosine	mimG	C15O5N5H19	349.1386	350.1459
N2,2â€²-O-dimethylguanosine	m2Gm	C12O5N5H17	311.1230	312.1303
N2,7,2â€²-O-trimethylguanosine	m2,7Gm	C13H21N5O5	327.1543	328.1616
N2,7-dimethylguanosine	m2,7G	C12O5N5H19	313.1386	314.1459
N2,7-dimethylguanosine cap (cap DMG)	m2,7Gpp(pN)	C12H17N5O11P2	469.0400	470.0473
N2,N2,2â€²-O-trimethylguanosine	m2,2Gm	C13O5N5H19	325.1386	326.1459
N2,N2,7-trimethylguanosine	m2,2,7G	C13O5N5H21	327.1543	328.1616
N2,N2,7-trimethylguanosine cap (cap TMG)	m2,2,7Gpp(pN)	C13H19N5O11P2	483.0556	484.0629
N2,N2-dimethylguanosine	m2,2G	C12O5N5H17	311.1230	312.1303
N2-methylguanosine	m2G	C11O5N5H15	297.1073	298.1146
N4,2â€²-O-dimethylcytidine	m4Cm	C11O5N3H17	271.1168	272.1241
N4,N4,2â€²-O-trimethylcytidine	m4,4Cm	C12O5N3H19	285.1325	286.1398
N4,N4-dimethylcytidine	m4,4C	C11O5N3H17	271.1168	272.1241
N4-acetyl-2â€²-O-methylcytidine	ac4Cm	C12O6N3H17	299.1117	300.119
N4-acetylcytidine	ac4C	C11O6N3H15	285.0961	286.1034
N4-methylcytidine	m4C	C10O5N3H15	257.1012	258.1085
N6,2â€²-O-dimethyladenosine	m6Am	C12O4N5H17	295.1281	296.1354
N6,N6,2â€²-O-trimethyladenosine	m6,6Am	C13O4N5H19	309.1437	310.1510
**N6,N6-dimethyladenosine**	**m6,6A**	**C12O4N5H17**	**295.1281**	**296.1354**
N6-(cis-hydroxyisopentenyl)adenosine	io6A	C15O5N5H21	351.1543	352.1616

N6-acetyladenosine	ac6A	C12O5N5H15	309.1073	310.1146
N6-formyladenosine	f6A	C11H13N5O5	295.0917	296.0990
N6-glycinylcarbamoyladenosine	g6A	C13O7N6H16	368.1080	369.1153
N6-hydroxymethyladenosine	hm6A	C11H15N5O5	297.1073	298.1146
N6-hydroxynorvalylcarbamoyladenosine	hn6A	C16O8N6H22	426.1499	427.1572
N6-isopentenyladenosine	i6A	C15O4N5H21	335.1594	336.1667
N6-methyl-N6-threonylcarbamoyladenosine	m6t6A	C16O8N6H22	426.1499	427.1572
**N6-methyladenosine**	**m6A**	**C11O4N5H15**	**281.1124**	**282.1197**
**N6-threonylcarbamoyladenosine**	**t6A**	**C15O8N6H20**	**412.1343**	**413.1416**
peroxywybutosine	o2yW	C21O11N6H28	540.1816	541.1889
preQ0base	preQ0base	C7O1N5H5	175.0494	176.0567
preQ1base	preQ1base	C7O1N5H9	179.0807	180.088
pseudouridine	Y	C9O6N2H12	244.0695	245.0768
Qbase	Qbase	C12O3N5H15	277.1175	278.1248
queuosine	QtRNA	C17O7N5H23	409.1597	410.1670
undermodified hydroxywybutosine	OHyWx	C18O8N6H24	452.1655	453.1728
uridine	U	C9O6N2H12	244.0695	245.0768
uridine 5-oxyacetic acid	cmo5U	C11O9N2H14	318.0699	319.0772
uridine 5-oxyacetic acid methyl ester	mcmo5U	C12O9N2H16	332.0856	333.0929
wybutosine	yW	C21O9N6H28	508.1918	509.1991
wyosine	imG	C14O5N5H17	335.1230	336.1303

Table S2. List of the 60 putative RNA adducts detected in the amphipods using *nLossFinder*. The table includes the *m/z* observed of the precursor ions in MS1 (MS1_mz) and of corresponding nucleobase adduct fragment ions in MS2 (MS2_mz), difference between the MS1 and MS2 ions (mz_diff, i.e., observed neutral loss), retention times in min (MS1_time, MS2_time), absolute intensities (MS1_int, MS2_int) and peak areas (MS1_Area, MS2_Area) for the respective MS1 and MS2 ions. The DIA window central *m/z* (DIA_mz) and the neutral loss error (nLoss_err) are also shown. The latter is expressed as Δppm between the neutral loss calculated (132.0423) and that observed. Six of the adducts were structurally identified (shown in Table 2), which are highlighted in bold.

MS1_mz	MS2_mz	mz_diff	MS1_time	MS2_time	MS1_int	MS2_int	MS1_Area	MS2_Area	DIA_mz	nLoss_err
255.1315	123.0891	132.0424	2.3	2.5	68296	1139	90104	3234	260	0.299
**258.1086**	**126.0660**	**132.0426**	**3.1**	**3.1**	**17273784**	**15340596**	**38534543**	**24569383**	**260**	**1.19**
**259.0925**	**127.0501**	**132.0425**	**4.8**	**4.8**	**6551218**	**2009248**	**14323235**	**4783573**	**260**	**0.625**
262.1393	130.0975	132.0418	8.9	8.9	10379	1931	24385	5141	260	1.96
262.1399	130.0975	132.0424	8.5	8.4	26030	749	38870	1399	260	0.232
262.1514	130.1083	132.0431	12.0	12.1	1235714	3962	2899991	5984	260	3.09
263.1241	131.0817	132.0424	4.4	4.5	17403	626	25482	1063	260	0.195
264.1449	132.1015	132.0434	7.8	7.8	24930	808	66319	3459	260	4.03
265.0403	132.9982	132.0421	2.2	2.2	48289	6622	81623	8827	270	0.722
265.1387	133.0970	132.0417	9.4	9.6	121006	610	311528	1053	270	2.35
266.0752	134.0326	132.0426	2.9	3.0	8600122	2947580	17400685	6642913	270	1.22
269.0330	136.9896	132.0434	10.5	10.4	135672	10874	276265	23630	270	4.11
**269.0881**	**137.0455**	**132.0425**	**4.8**	**4.8**	**8957064**	**2934871**	**19187032**	**6612468**	**270**	**0.866**
271.0932	139.0503	132.0429	5.7	5.6	23329	4831	47057	12068	270	2.31

273.1082	141.0657	132.0425	9.1	9.0	7802	584	14313	2425	270	0.648
273.1082	141.0657	132.0425	8.7	8.7	10075	715	10075	715	270	0.693
279.1588	147.1171	132.0417	14.0	13.8	23685084	24767	40133556	202866	280	2.03
281.0538	149.0123	132.0415	6.5	6.6	274362	1805	617652	2301	280	2.87
**282.1194**	**150.0775**	**132.0419**	**6.8**	**6.9**	**81553792**	**39150732**	**229245392**	**107130679**	**280**	**1.27**
283.1037	151.0614	132.0423	10.9	11.0	181931	114098	430982	223732	280	0.149
284.0895	152.0474	132.0421	5.1	5.0	352460	79072	856417	146668	280	0.704
286.1032	154.0611	132.0422	5.1	5.0	413975	68763	977744	129013	290	0.382
287.1236	155.0814	132.0423	4.5	4.4	22919	703	108620	1787	290	0.158
287.1968	155.1544	132.0424	9.7	9.7	13933	7767	56043.9	27338	290	0.235
**296.1355**	**164.0933**	**132.0422**	**10.2**	**10.2**	**1246454**	**549570**	**2338938**	**1412519**	**300**	**0.494**
**298.1150**	**166.0723**	**132.0427**	**7.0**	**6.9**	**565791**	**328187**	**1115574**	**767049**	**300**	**1.24**
298.1765	166.1343	132.0422	5.3	5.5	30968	1189	85075	2855	300	0.377
300.1305	168.0884	132.0421	6.2	6.2	72807	772	190554	1554	300	0.568
301.1142	169.0719	132.0423	4.7	4.8	85424	25576	180009	62209	300	0.090
301.2123	169.1698	132.0425	12.7	12.9	1832814	937	4462608	1679	300	0.623
303.1556	171.1130	132.0426	11.0	10.8	79222	10036	217883	29313	300	1.05
304.1579	172.1159	132.0421	9.0	9.1	10611	2086	19971	3771	300	0.778
306.0807	174.0387	132.0420	5.1	5.1	1889788	912689	5112179	2427312	310	0.861
307.1289	175.0867	132.0421	10.4	10.4	72422	574	154458	574	310	0.550

307.1514	175.1077	132.0437	10.1	10.2	11927	3818	21933	8436	310	4.59
309.2061	177.1639	132.0422	13.8	14.1	11039837	6135	19985107	38989	310	0.320
312.1301	180.0878	132.0423	8.5	8.5	295315	180800	529388	372161	310	0.106
315.1260	183.0846	132.0415	8.1	8.1	34621	33159	52426	66901	320	2.64
317.1467	185.1034	132.0433	8.8	8.9	101618	821	203317	3100	320	3.14
318.1659	186.1234	132.0426	3.3	3.2	6329985	16503	20071933	52025	320	0.890
320.0971	188.0543	132.0429	7.1	7.0	21852	16890	33192	36696	320	1.76
321.1671	189.1234	132.0437	8.8	8.9	110018	44803	278887	148014	320	4.23
323.0644	191.0225	132.0419	6.6	6.5	22663	2568	41282	4757	320	1.25
324.1653	192.1215	132.0438	5.1	5.2	12203	7130	24100	13242	320	4.60
325.1394	193.0972	132.0422	7.3	7.2	72231	3498	330361	9194	330	0.252
325.2009	193.1585	132.0424	13.8	14.0	3221547	2238	7829550	19706	330	0.349
326.1181	194.0755	132.0426	7.4	7.3	30837	9536	286628	119707	330	0.991
327.1552	195.1130	132.0422	7.2	7.3	17666	463	68619	463	330	0.417
327.2029	195.1606	132.0423	2.8	3.0	119572	4012	288886	12540	330	0.00291
329.1567	197.1149	132.0418	12.8	12.9	763371	5001	2824967	15195	330	1.37
332.0304	199.9882	132.0422	6.5	6.5	13348	5241	31887	11724	330	0.303
338.1348	206.0928	132.0419	5.4	5.7	365876	30019	1182026	137500	340	1.05
339.1775	207.1362	132.0413	13.5	13.7	1090047	2090	5173139	7675	340	3.08
340.1255	208.0829	132.0426	7.7	7.7	1284218	765946	11226529	6708865	340	1.00

341.1228	209.0799	132.0429	7.6	7.7	23063	14493	134438	114890	340	1.80
341.1285	209.0864	132.0422	7.7	7.7	179578	65527	1447314	548979	340	0.422
341.1710	209.1286	132.0425	11.2	11.3	70075	774	562812	2523	340	0.487
342.2637	210.2213	132.0424	13.7	13.9	186671	2936	657227	12502	340	0.217
345.0605	213.0166	132.0439	6.5	6.5	196509	3049	449520	4608	350	4.57
349.9491	217.9069	132.0422	2.7	2.8	103152	5396	103152	9028	350	0.194
